# A novel mutation in *GJA3* associated with congenital Coppock-like cataract in a large Chinese family

**Published:** 2012-07-26

**Authors:** Lu Zhang, Xin Qu, Sheng Su, Linan Guan, Ping Liu

**Affiliations:** 1 Eye Hospital, the First Affiliated Hospital, Harbin Medical University, Harbin, China; 2 The Third Affiliated Hospital, Harbin Medical University, Harbin, China

## Abstract

**Purpose:**

To identify the potential pathogenic mutation over five generations of a Chinese family with congenital Coppock-like cataracts (CCL).

**Methods:**

We investigated five generations of a Chinese family affected with CCL. The family resides in a relatively isolated region of northern China. Peripheral blood samples were collected from all of the family members, and genomic DNA was then extracted from the blood samples. A genome-wide linkage scan was performed using about 400 microsatellite markers. Two-point LOD (linkage odd disequilibrium) scores (Z) were calculated using the LINKAGE programs (ver. 5.1). Cyrillic software processed the resulting haplotypes. Mutation detection was performed in the candidate gene by direct sequencing.

**Results:**

The maximum LOD score was obtained at marker D13S175 (lod score [Z_max_]=5.90; recombination fraction [θ]=0.0). Haplotype analysis traced the disease gene to a 6.99-cM interval bounded by D13S1316 and D13S1275 on chromosome 13q12.11. Direct sequencing of the candidate gene *GJA3* (gap junction protein alpha-3) revealed a c.427G>A transition in exon 2 of *GJA3* that co-segregated with the cataract in the family members and was not observed in 100 control patients. This single-nucleotide change resulted in the substitution of a highly conserved glycine by arginine (G143R).

**Conclusions:**

The present study identified a novel mutation in *GJA3* that causes CCL. As the first report to relate p.G143R mutation in *GJA3*, it expands the mutation spectrum of *GJA3*. Our report is the first in identification of the mutation of *GJA3* in the cytoplasmic-loop domain. This mutation is associated with multiple members of a five-generation family with congenital CCL.

## Introduction

A congenital cataract is a clinically and genetically heterogeneous lens disorder that typically appears as a sight-threatening trait during childhood, accounting for one-tenth of the cases of childhood blindness [1]. Approximately half of all congenital cataract cases are inherited either in isolation or as part of a syndrome of ocular or systemic abnormalities [2]. All three classical forms of Mendelian inheritance have been associated with nonsyndromic cataracts. However, the majority of families with a history of congenital cataracts show an autosomal dominant inheritance pattern. So far, genetic linkage studies of around 180 families worldwide have mapped at least 35 independent loci and identified mutations in over 20 genes for phenotypically diverse forms of primary cataract involving total, nuclear, lamellar/zonular, sutural, and polar and/or sub-capsular lens opacities [3]. The molecular characterization of different phenotypes is important for the identiﬁcation of the various elements involved in lens opaciﬁcation.

The Coppock cataract refers to a pulverulent disc-like opacity involving the embryonal and fetal nucleus. It was ﬁrst reported by Nettleship et al. [4] in 1906. The Coppock cataract was mapped to chromosome 1q and a mutation identiﬁed in *GJA8* (gap junction protein alpha-8) [5,6]. Morphologically similar cataracts are referred to as the Coppock-like cataract (CCL). One CCL was mapped to chromosome 2q and a mutation identiﬁed in *CRYGC* (gammaC-crystallin) [7]. Another CCL was mapped to chromosome 22q and a mutation identiﬁed in *CRYBB2* (beta-crystallin B2) [8].

Here we have mapped autosomal dominant congenital CCL segregating in a Chinese family to chromosome 13q and identified a novel missense mutation in the gene for gap-junction protein alpha-3 (*GJA3*), or the connexin46 gene (Cx46).

## Methods

### Clinical evaluation and DNA specimens

**Figure 1:**
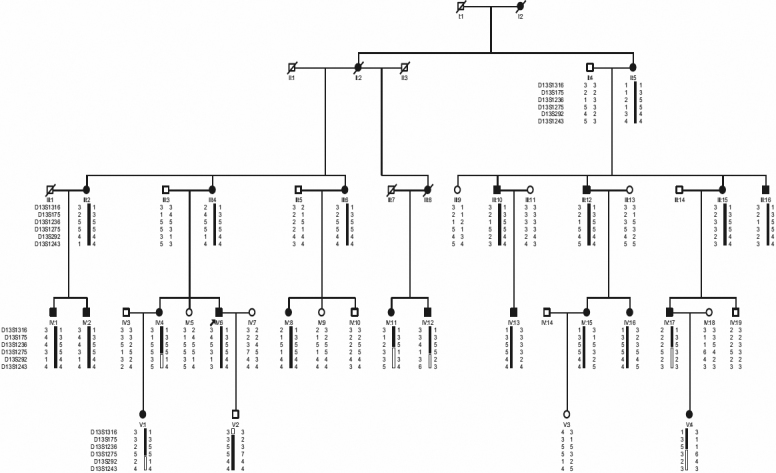
Pedigree and haplotype of the family with autosomal dominant congenital Coppock-like cataract. A pedigree is shown with the haplotype analysis of the Chinese family with cataracts, which shows the segregation of 6 microsatellite markers on chromosome 13q in descending order from the centromere. Squares and circles represent males and females, respectively. Black and white symbols denote affected and unaffected individuals, respectively.

We identified a five-generation Chinese family with autosomal dominant CCL in the absence of other ocular or systemic abnormalities. The family resides in a relatively isolated region of northern China. All participants provided informed consent in accordance with the Declaration of Helsinki. Forty-six individuals of this 150-member family participated in the study, twenty-four affected and twenty-two unaffected (Figure 1). Family members were considered “affected” by a history of either cataract extraction or ophthalmologic examination, which included visual acuity testing, slit lamp examination, intraocular pressure measurement, and fundus examination with dilated pupils. Phenotypes were documented using slit lamp photography. Peripheral blood was collected and genomic DNA was extracted from blood lymphocytes leukocytes using a QIAampDNA Blood Mini Kit (Qiagen, Hilden, Germany).

### Genotyping and linkage analysis

We conducted a genome-wide linkage scan based on a set of dinucleotide repeat microsatellite markers spaced at approximately 10 cM intervals using an ABI PRISM Linkage Mapping Set (ver. 2.5; Applied Biosystems, Foster City, CA). Additional microsatellite markers for the positive region were selected for precise targeting. A “touchdown” polymerase chain reaction (PCR) was performed in a 5 μl reaction volume containing 20 ng genomic DNA, 1 µl 10× PCR buffer, 7 mM MgCl_2_, 0.2 mM dNTPs, 0.3 U HotStar Taq DNA polymerase, and 0.05 µM microsatellite markers. After an initial denaturation period of 12 min at 95 °C, 14 cycles were performed at 95 °C for 30 s, 63–56 °C for 30 s (with a 0.5 °C decrease at each step), and 72 °C for 1 min. Thirty cycles were performed at 95 °C for 30 s, 56 °C for 30 s, and 72 °C for 1 min followed by an extension at 72 °C for 10 min and a final hold at 4 °C. The PCR products were pooled on the basis of size (Genescan-400HD ROX; Perkin Elmer, Foster City, CA), denatured at 95 °C for 1 min, and electrophoresed in a 96 capillary automated DNA analysis system (MegaBACE 1000; Amersham, Freiburg, Germany). The results were analyzed by a Genetic Profiler (ver. 1.5; Amersham). Two-point logarithm of the odds (LOD) scores (Z) calculated using the MLINK sub-program from the LINKAGE (ver. 5.1) package of programs. A gene frequency of 0.0001 and a 95% penetration were assumed for the cataract locus. LOD scores were calculated at recombination fractions (θ) of 0, 0.1, 0.2, 0.3, 0.4, and 0.5.

### Mutational analyses

One known candidate gene, *GJA3*, is located within the physical region defined for the cataract on chromosome 13q12.11. We systematically sequenced the *GJA3* gene in two affected and two unaffected members of the family using specific primers (Table 1). Genomic DNA was PCR amplified, purified, and sequenced directly using dye-terminator chemistry. The purified PCR products were sequenced on both DNA strands using an ABI 3100 sequencer (Applied Biosystems). After identifying a missense mutation in exon 2 of *GJA3*, all of the family members and 100 unrelated normal individuals were screened.

**Table 1:** PCR primers for mutational screening of *GJA3*.

**Exon 2**	**Strand**	**sequence (5′-3′)**
2a	Sense	CCATCCCAGTACCATCCAG
** **	Antisense	TCTCTTCAGCTGCTCCTCCT
2b	Sense	AGAACGTCTGCTACGACAGG
** **	Antisense	CCTGCTTGAGCTTCTTCCAG
2c	Sense	CGAGCTGAAGCCGCTCTA
** **	Antisense	CTGCCGGGTAAGCCTTGA
2d	Sense	CGCGGACTTCAAACTGCTA
** **	Antisense	TCTATCTGCTGGTGGGAAGTG

## Results

### Clinical findings

**Figure 2:**
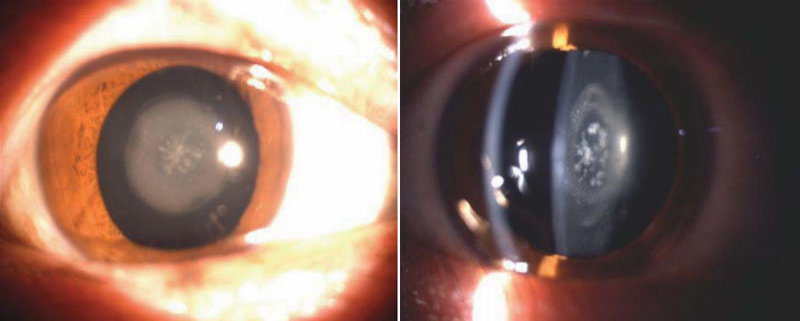
Slit lamp photographs of the eye of affected individuals. Slit lamp photographs showed that bilateral granular opacity in the center of the lens of every affected member.

Autosomal dominant inheritance was supported by the presence of affected individuals in each generation via male-to-male transmission, with approximately equal numbers of affected males and females (Figure 1). The affected individuals presented with bilateral granular opacity in the center of the lens (Figure 2). Ophthalmoscopic examination showed that the cataract appeared as a circular spotted disc in the center of the lens. The disc observed by slit lamp exhibited small spotted or granular opacity. The Y-suture could not be definitely identified but the opacity appeared only to be involved in the embryonic nucleus. Thus, affected individuals in the family were diagnosed affected congenital CCL. Most patients experienced decreased visual acuity around 7–8 years old. Some of those affected required cataract surgery during childhood, occasionally in the 40s.

### Linkage analysis

By following the exclusion of known chromosomal regions related to Coppock cataract and other chromosomal regions, we obtained significant evidence of linkage for marker D13S1236 (Z=4.49, θ=0). The maximum two-point lod score (Z_max_) of 5.90 was obtained at marker D13S175 with recombination θ=0.00. The adjacent markers (D13S1316) also showed lod scores greater than 3.0. The results of the two-point lod scores are summarized in Table 2.

**Table 2:** Two-point LOD scores for linkage analyses.

** **	** **	**LOD score at θ=**						** **	** **
**Markers**	**Position (Mb)**	**0**	**0.1**	**0.2**	**0.3**	**0.4**	**0.5**	**Z_max_**	**θ_max_**
D13S1316	20.6	5.55	4.57	3.54	2.44	1.26	0.00	5.55	0.0
D13S175	20.8	5.90	5.18	4.11	2.83	1.39	0.00	5.90	0.0
D13S1236	22.6	4.49	3.67	2.79	1.85	0.85	0.00	4.49	0.0
D13S1275	22.9	−8.38	0.46	0.68	0.52	0.20	0.00	0.68	0.2
D13S292	24.1	−14.26	0.55	0.92	0.77	0.37	0.00	0.92	0.2
D13S1243	24.8	−8.40	0.33	0.62	0.56	0.34	0.00	0.62	0.2

### Haplotype analysis

Haplotypes of the family were constructed with six microsatellite markers (Figure 1). Six crossovers occurred in the affected individuals IV:4, IV:11, IV:12, and IV:17, V:1 and V:4 defined the distal border of the region. Marker D13S1316, the first marker of chromosomal 13q, defined the proximal border of the region. The diseased haplotype shared by all affected members was identified. The results of both the linkage and haplotype analyses situated the diseased gene in a 6.99 cM region bounded by D13S1316 and D13S1275 at 13q12.11, which contains *GJA3*. All affected individuals had an affected parent and none of the unaffected individuals carried the diseased haplotype.

### Mutational analysis

**Figure 3:**
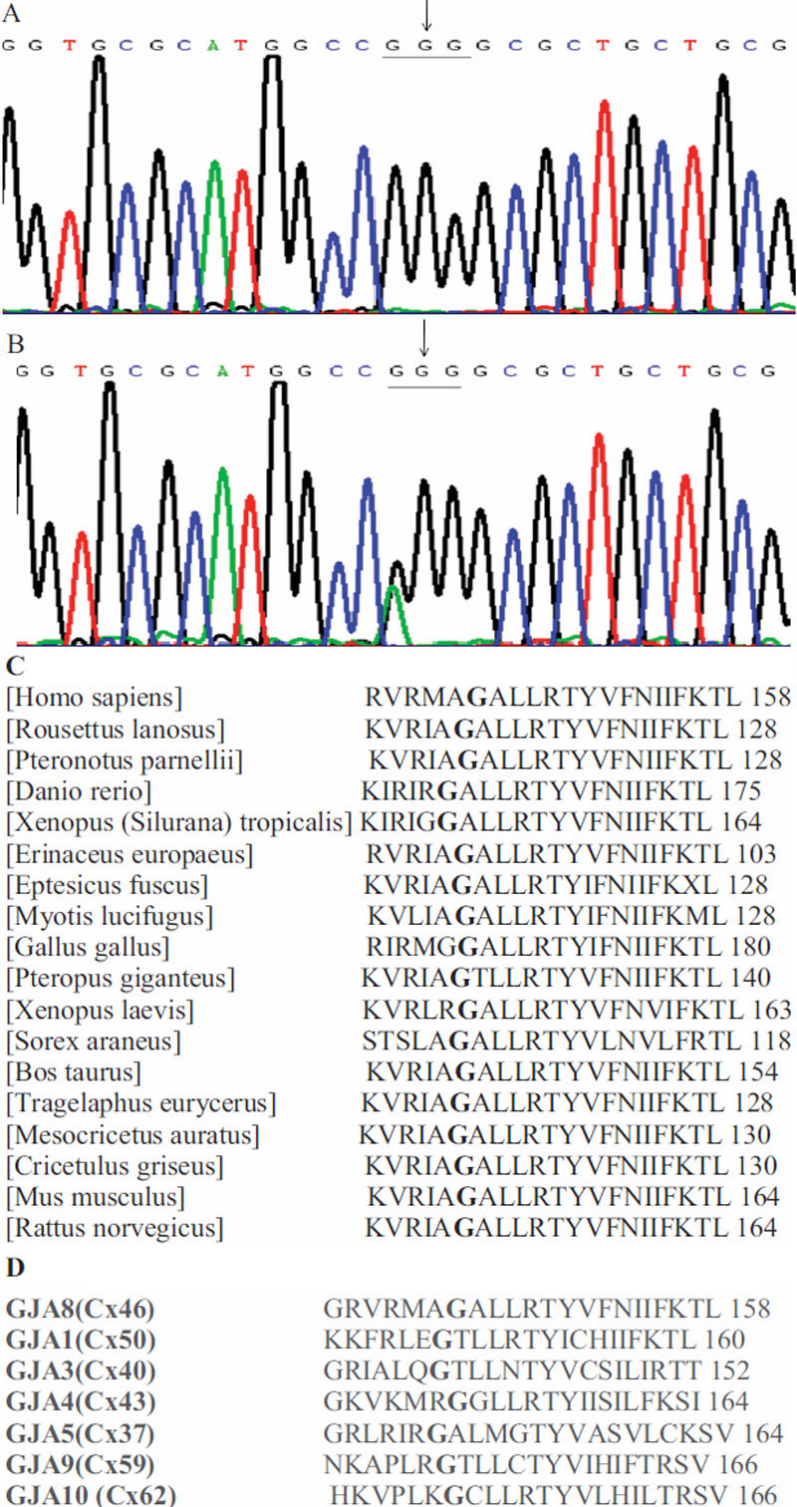
Mutational analysis of *GJA3*. **A**: Sequence chromatograms of the wild-type *GJA3* allele, showing that the wild-type gene encodes Glycine residue (GGG). **B**: Sequence chromatograms of the mutant allele, showing a G→A transition that substituted Arginine residue (AGG). **C**: Glycine at position 143 of Cx46 protein is highly conserved across animal species. **D**: Amino acid residue Gly-143 is conserved in various types of human gap junction protein.

Direct sequence analysis of *GJA3* identified a c.427G>A transition in exon 2. This single-nucleotide change resulted in a missense mutation at codon 143, changing a glycine residue to arginine residue (G143R). The cosegregation of the c. 427G>A transition was only found in affected family members (Figure 3B) and was absent both in the unaffected family members and the control group (Figure 3A). This result strongly suggests that the G143R substitution was a causative mutation rather than a benign single nucleotide polymorphism (SNP) in linkage disequilibrium with the cataract.

## Discussion

The lens consists of three parts: the outermost lens capsule, the interior lens fiber that forms the bulk of lens, and the lens epithelium, which is located between other two structures and only presented on anterior side. The fiber cells are linked with each other and connected with cells at the lens surface via gap junction channels, a large intercellular communication network. Because of a lack of blood supply in the lens, gap junction coupling largely contributes to lens homeostasis and the maintenance of transparency [9,10].

Gap junctions are clusters of transmembrane channels that connect two adjacent cells and allow small molecules (M_r_≤1 kDa), such as ions, metabolites and second messengers, to pass from cell to cell [11]. A gap junction channel is formed by two hemichannels, also called connexons, from two neighboring cells that are aligned with each other. Each connexon consists of six connexins, which are a family of membrane protein containing twenty-one members in humans [12]. Connexin consists of four conserved transmembrane domains, two extracellular loop domains, one intracellular loop domain, and cytoplasmic NH_2_- and COOH-terminal domains. Compared with conserved transmembrane domains, extracellular loop and cytoplasmic NH_2_-terminal, intracellular loop and COOH-terminal domains are highly variable domains and are subjected to post-translational regulation (i.e., phosphorylation) [13,14].

Cx46 and Cx50 (connexin50) are predominantly expressed in lens fiber cells. Cx46 plays a critical role in coupling of fiber cells, especially in mature fiber in central core of the lens. Deletion of the *GJA3* (Cx46) gene leads to severe nuclear cataracts in mice [15]. The mutations of *GJA3* (Cx46) and GJA8 (Cx50) are directly linked to human congenital cataracts [9]. Interestingly, most of fiber connexin mutations identified are located in transmembrane and extracellular loop domains. So far, over 20 cataract-associated mutations of Cx46 have been identified [16]. However, only two mutations, N63S and fs380, have been characterized. Both mutations show the loss of gap junction coupling [17].

In the present study, we identified a c.427G>A transition in exon 2 of *GJA3*, which caused autosomal dominant congenital CCL. This single-nucleotide change led to the replacement of neutral amino acid residue glycine to positive charge residue arginine. The glycine at position 143 is highly conserved across various species (Figure 3C) and human connexins (Figure 3D). This mutation is located in the cytoplasmic loop domain of Cx46. Over a decade or so, more than 20 different mutations of Cx46 including missense and frame-shift mutation have been identified as being related to congenital cataracts in various family origins across continents [9,16]. However, the majority of the mutations occur in the extracellular loop and transmembrane domains. Recently, a mutation V139M in the intracellular loop domain was identified in a 76-year-old male Chinese patient; however, this patient had no family history of congenital cataracts [18]. Our report is the first to identify of the mutation of Cx46 in the cytoplasmic loop domain. This mutation is associated with multiple members of a five-generation family with congenital CCL.

In conclusion, a novel mutation of the *GJA3* (Cx 46) gene was identified in a five-generation Chinese family with dominant congenital CCL. These findings thus expand the mutation spectrum of *GJA3*.
